# Output order effects in autobiographical memory in old age: further evidence for an emotional organisation

**DOI:** 10.3758/s13421-022-01312-3

**Published:** 2022-05-31

**Authors:** Daniel Zimprich, Lisa Nusser

**Affiliations:** Developmental Psychology, Institute of Psychology and Education, Ulm University, Albert-Einstein-Allee 47, 89081 Ulm, Germany

**Keywords:** Autobiographical memory, Autoregressive effects, Output order, Emotional valence, Emotional intensity, Mood, Mixed-effects model

## Abstract

It is generally accepted that autobiographical memories (AMs) are organised in associative networks. While both thematic and temporal similarity have been examined as connections among AMs, in the present study we focused on both the positive and negative emotional intensity of events as a possible link among AMs. To do so, we investigated whether the output order with which AMs elicited by cue words were reported can be accounted for emotional intensity of adjacent AMs. Data come from 94 older adults (*M* $=$ 67.14; *SD* $=$ 6.17) who reported 30 AMs in response to neutral cue words. Positive and negative emotional intensity of AMs were assessed on two separate scales (happiness and sadness). The output order was modeled based on a dual mixed-effects autoregressive model, where the strength of the autoregressive effect indicates how much the emotional intensity of an AM can be predicted by the emotional intensity of the previously reported AM. Results show that there were significant autoregressive effects for both the happiness and sadness ratings (accounting for 4% of variance). We also observed cross-over effects, such that the happiness rating of an AM was predicted by the sadness rating of the previously reported AM (and vice versa). Moreover, we found individual differences in the strength of the autoregressive effects. For the sadness ratings, these individual differences tended to be related to the participant’s mood state, particularly so during the first output positions. Together, these findings demonstrate that there is a substantive effect of emotional intensity on the output order with which AMs are reported—even when elicited by cue words. Based on the premise that the output order of AMs informs about the organisation of autobiographical memory, our results highlight the role of emotional associations among AMs in old age.

## Introduction

There is broad consensus that autobiographical memories (AMs), defined as the recollection of experiences from ones’s personal past (Fivush, 2011), are not stored as isolated mental representations. Rather, AMs are represented in memory as a collection of attributes that form a network of associations, with this network possibly encompassing several hierarchical levels (Conway, 2005; Conway & Pleydell-Pearce, 2000). Such a network based on associations among attributes of AMs can assumed to find expression—at least in part—in the recall pattern, that is, the order with which people retrieve AMs. Thus, investigating the recall order may open a window into the associations among AMs and help to reveal the organisation of autobiographical memory (Mace, 2014)—much as it did in episodic memory research (e.g., Kahana, 1996; Kurtz & Zimprich, 2014).

Studies examining the recall order of autobiographical memories have demonstrated that associations among AMs are formed based on similar characteristics of the events being described, such as time of the event (e.g., Brown & Schopflocher, 1998; Moreton & Ward, 2010; Nusser & Zimprich, 2021), themes, persons involved, or activities (e.g., Mace & Hall, 2018). More abstract features of AMs may also play a role, such as identity motives or needs related to the events (Woike, Lavezzary, & Barsky, 2001; Philippe, Koestner, Beaulieu-Pelletier, Lecours, & Lekes, 2012). Recent studies also provided evidence that emotion (i.e., the emotional valence of an AM) may associate AMs (Nusser & Zimprich, 2021; Philippe, Koestner, Lecours, Beaulieu-Pelletier, & Bois, 2011; Philippe, Lecours, & Beaulieu-Pelletier, 2009). While there is ample evidence that emotional attributes represent an organising principle in episodic memory (e.g., Talmi et al., 2019), studies examining the role of emotions in the organisation of autobiographical memory are scarce. While there are studies that have demonstrated how *one* AM is emotionally associated with *several* other AMs (e.g., Philippe et al., 2009, 2011), what is missing is a more thorough investigation of the impact of emotion when it comes to longer sequences of AMs reported by individuals. The overarching goal of the present research, therefore, was to examine the emotional organisation of autobiographical memory based on the output order of AMs. Different from previous studies, we modelled the strength of the emotional association between adjacently reported AMs in a sequence of 30 AMs reported by 94 older participants. More specifically, we employed a dual mixed-effects autoregressive model to examine whether the happiness and sadness ratings of an AM affected the happiness and sadness of the AM reported next.

### Emotional ordering in autobiographical memory

The information representing a single AM in memory is distributed over multiple attributes, features, elements, or dimensions (these terms are used interchangeably in the literature). One important attribute of AMs is the emotion that the event stored in memory elicited when the event happened. In line with this, the relation between autobiographical memories and emotion has been examined in a number of studies (see, e.g., Holland & Kensinger, 2010; Zimprich & Wolf, 2018). However, the organising role of emotion in autobiographical memory received comparatively little attention. One reason for this may be that current models of autobiographical memory rarely consider the role of emotions for the organisation of AMs and, if so, in a relatively simplified manner (Luminet, 2021). For example, in the hierarchical model of the organisation of autobiographical memory proposed by Conway and Pleydell-Pearce (2000), emotions are not explicitly localized. In supplementing this model, some researchers suggested that emotional features of an AM may be considered as one type of event-specific knowledge, which together with other phenomenological details would then be embedded at the lowest hierarchical level in the model (Holland & Kensinger, 2010).

On the other hand, based on the assumption that recall order reflects the organisation of autobiographical memory, there are studies demonstrating that emotion can play a role. For instance, Philipp and colleagues (2009), using the event-cuing technique developed by Brown and Schopflocher 1998), asked participants to report a personal memory that a sad film excerpt made them spontaneously think about, which was then used as the cue for recalling related memories. In this study, participants were explicitly asked to recall other personal memories related to the initial memory they had just described. The authors found positive correlations ($r = .18$ to $r = .39$) between the positive (negative) valence of the initial memory and the number of positive (negative) subsequent memories reported. Similarly, Philippe et al. (2011) found a significant correlation between the valence of a loss-related memory and the valence of associated memories ($r = .37$). Also, Wright and Nunn (2000) demonstrated that autobiographical memories within event clusters, containing one initial memory and six additional memories cued by that initial memory, were more similar with respect to their emotional valence compared to events from different clusters.

To account for these results, Philippe et al. (2009) proposed the *emotional memory networks theory*, which states that every time an AM with a specific emotional valence is activated, activation automatically spreads to other AMs similar in emotional valence, leading to an increase in the activation of the latter. This theoretical approach resembles traditional spreading activation models such as the associative network theory suggested by Bower (1981), in which emotions are represented as nodes within a network .^1^ Bower (1981) postulated that emotion-related aspects (e.g., a mood state) automatically prime or pre-activate representations linked to that emotion.^2^ Based on the spreading activation assumption, an emotional ordering of AMs during recall may thus be explained by a recall process where the retrieval of an AM raises the activation levels of emotionally similar AMs, one of which is then selected for recall. Of course, apart from the emotional attributes of an AM, other attributes become activated as well (e.g., temporal information, see Moreton and Ward, 2010). However, emotional attributes may form relatively strong associations among AMs.

A different, but related theoretical perspective suggested in conjunction with episodic memory (see Talmi et al., 2019) focuses on context, which describes a mental representation that captures both external characteristics of a situation (such as the environment in which an event occurs) and also the concurrent internal state of an individual (e.g., being in a happy mood when an event occurs). During experiencing an event, not only external characteristics of the event become attributes of an AM, but also features of the concurrent internal context. During later recall (and in a different external and internal context), an AM might alter the current internal context of an individual in that a retrieved AM is assumed to impart its emotional attributes to the internal state—as well as other attributes of the AM do. More broadly, it is assumed that the context retrieved from an AM (i.e., the entirety of its attributes) is used to update the current context (cf. Long, Danoff, & Kahana, 2015). The role of context is central because it is assumed that the current context serves as the retrieval cue for the recall of subsequent AMs because the current context activates the representations of those AMs similar in context (e.g., Smith, 2007). Depending on the amount of similarity, an AM is selected, which then itself updates the current context—and the process recurs (cf. Howard & Kahana, 2002).

A possible limitation of the event-cuing technique used in previous studies to examine emotional order effects is that the experimental procedure with the explicit request of recalling *related* AMs in response to an initial AM may have increased the probability of recalling (emotionally) similar AMs. Essentially, these results show that participants are able to retrieve AMs similar (or related) to an initial AM when instructed to do so. That is, the external validity of this type of experiments and, thus, the generalisability of findings may be reduced (e.g., Lucas, 2003)—particularly with respect to a naturally occurring recall order of AMs. In a similar vein, the event-cuing technique focuses on whether *one* initial AM is associated with *multiple* AMs. To establish an emotional order effect, however, it would appear more appropriate to examine whether sequentially retrieved AMs are each associated with their respective predecessors. Using one initial AM to cue all other AMs ignores the proposition that each recalled AM functions as a cue for the retrieval of the next AM (cf. Mace & Clevinger, 2019). Therefore, in the present study, we decided to elicit autobiographical memories using 30 emotionally neutral cue words (cf. Nusser & Zimprich, 2021). By doing so, we wanted to avoid that participants purposively associate one autobiographical memory with the next. Moreover, our goal was to examine whether an emotional order during recall also emerges even when participants link each reported autobiographical memory with a cue word.

The process of recalling a sequence of AMs can be seen as a fluid movement from memory to memory, such that the output resulting from the retrieval process resembles an associative chain: Each recalled AM is linked to its neighbours. In this way, retrieval of the first AM facilitates the retrieval of the second, and the second facilitates retrieval of the third, and so forth. Note that, unlike theories of sequence memory (e.g., Lewandowsky & Murdock, 1989), we do not assume that AMs are *stored* in an associative chain in memory, but that the specific sequence of AMs recalled by a participant minimally implies that each AM is connected to its predecessor and its successor. Statistically, such an associative chaining process during the output of AMs corresponds to a first-order autoregressive model, which specifies that an outcome variable depends on the previous value of the outcome variable itself (plus a stochastic error term). In accordance with this, Nusser and Zimprich (2021) used a mixed-effects autoregressive model to examine emotional order effects in the recall of AMs. Core of the model was the autoregressive part, where the emotional valence of each AM was regressed on the emotional valence of the previously reported AM. In a sample of 117 older adults, the authors found a significant first and second order autoregressive effect of emotional valence, which, in addition, differed reliably between persons. These findings indicate that individuals exhibited a “carry-over” effect of the emotional valence of the current AM into the next reported AM. Moreover, some individuals showed a strong emotional order effect, whereas others even showed a negative, that is, reversed, emotional order effect.

If emotional features of one AM trigger the recall of the next AM, a straightforward question is whether this effect is stronger for AMs with more intense emotions. A distinction of emotions according to their valence is but one way to characterize emotional attributes of AMs, whereas a more fine-graded view would also take into account the intensity of emotions (Holland & Kensinger, 2010; Wolf, Pociunaite, Hoehne, & Zimprich, 2021). As Talarico and colleagues (Talarico, LaBar, & Rubin, 2004), for example, have shown in a series of experiments, emotional intensity is a significant predictor of the vividness of an AM, the amount of reliving an AM, and visceral reactions to the AM. Moreover, the proportion of variance accounted for in these variables by emotional intensity was greater than by of emotional valence. Based on these findings, one may conjecture that the recall of an emotionally more intense AM leads to a higher likelihood of retrieving an emotionally similar AM. Either by assuming that more activation is elicited by a more intense emotion—rendering other attributes of both the current AM and those AMs associated to it relatively less decisive in selecting the next AM for recall. Or, in terms of context models (e.g., Smith, 2007), based on the assumption that a more intense AMs imparts its emotional attributes more strongly to the current internal state, which serves as the retrieval cue for the next AM. Therefore, we hypothesize that the emotional order effect not only holds for emotional valence (Nusser & Zimprich, 2021), but also for the emotional intensity of AMs.

It is unlikely that an emotional order effect in recalling autobiographical memories is identical for all individuals. Accordingly, one would expect that there are between-person variables that lead to individual differences in emotional order effects. Most prominently, current mood of an individual may affect which AMs are retrieved. A number of studies have shown that mood influences the accessibility of memories in the sense that AMs congruent in valence with one’s present mood are more likely to be retrieved (e.g., Sakaki, 2007)—a phenomenon known as mood-congruent memory (see Barry, Naus, & Rehm, 2004, for a review). For instance, Miranda and Kihlstrom (2005) showed that AMs retrieved by participants in a positive mood were more positive than those retrieved by participants in a negative mood. Similarly, in a study conducted by Berntsen (2002), participants’ mood states were less positive after the recall of the most shocking experience from their life. In addition, subsequent retrieval of word-cued memories resulted in more negative AMs and fewer positive AMs. A more recent version of the associative network theory (Bower, 1981), the affect infusion model (Forgas, 1995), attempts to more clearly specify under which conditions the influence of mood on memory is expected to be stronger. It holds that mood-congruence effects are hypothesized to arise particularly in situations in which an individual must *generate* a response—such as in free recall or, transferred to the current research, in the recall of AMs elicited by cue words. The available empirical evidence with respect to episodic memory is mostly in line with this expectation (see Bower & Forgas, 2000).

What has not been examined to date is whether the effect of mood is constant across sequentially retrieved AMs or whether it changes across the recall process. From the perspective of context models, one might expect that the influence of mood gradually attenuates due to a drift in (internal) context produced by the emotional attributes of AMs already recalled (cf. Talmi et al., 2019). That is, because each retrieved AM imparts its emotional (and other) attributes to an individual’s current internal context, context successively moves away from the initial internal context representing the individual’s mood at the beginning of the recall process. As an objection, one might argue that an individual could be inclined to recall only AMs that correspond to her or his current mood, such that a context drift would not occur. This is, however, unlikely once longer sequences of AMs are examined, because the selection of the next AM for recall is a process that also entails a probabilistic component (cf. Unsworth, Brewer, & Spillers, 2014). Moreover, other, non-emotional attributes of AMs also affect recall, such that an AM selected due to its, for example, thematic overlap with the previous AM would still change the internal context via its emotional features. Consequently, one might conjecture a bidirectional influence between mood, which promotes recall of emotionally congruent AMs, and the emotional attributes of a retrieved AM, which in turn influence current mood. However, because a drift of internal context is assumed to take longer, that is, changes gradually over time—emotional features of an AM affect internal context, but do not completely determine it—we hypothesize that current mood might particularly influence an emotional order effect during the first output positions. For later output positions, the internal context is expected to have moved away from the initial mood state, such that an effect of the latter attenuates.

### Aims of the present study

The major goal of the present study was to examine whether the output order of AMs elicited by cue words in old age can be explained by the emotional intensity of the events reported in the AMs. Our focus on old age was motivated by the following rationale. Older adults (compared to younger adults) have access to a much more varied pool of life events kept in autobiographical memory. As a consequence, one may expect that, in principle, their AMs differ more regarding many attributes, e.g., emotionality. At the same time, because some (but not all) AMs remain accessible across years or decades in old age, a selective retention mechanism might come into play that is based on the distinctiveness of an event. One factor that contributes to the distinctiveness of an events is their emotional valence and their emotional intensity. A second aim of the present study was to replicate and extend the emotional output order effect in old age reported by Nusser and Zimprich (2021). Different from their approach, in the present study we considered the output order of AMs with respect to two outcome variables (happiness and sadness ratings of AMs) simultaneously. As a consequence, autoregressive effects could be examined both *within* outcome variables (e.g., how does the happiness rating of the previously recalled AM affect the happiness rating of the current AM?) and *across* outcome variables (e.g., how does the happiness rating of the previously recalled AM affects the sadness rating of the current AM?). This allowed us to examine whether an output order for happiness also exerts influence on the output order for sadness and vice versa. In addition, the effect of current mood on happiness and sadness ratings and their respective emotional order was investigated based on the assumption that, across output positions, a mood-congruency effect may be observed, which, possibly, would be most pronounced during the first output positions. Finally, we expected mood to amplify the emotional order effect especially during the first output positions.

To account for the dependency inherent in the data analysed in the present study (every participant reported up to 30 AMs), random intercepts and random autoregressive effects were modeled for both outcome variables (see Modeling Approach), leading to a dual mixed-effects autoregressive model. Moreover, age and sex of participants were included as predictor variables. Although the positivity effect found in older adults (e.g., Ros & Latorre, 2010) and results indicating that women tend to recall more emotional AMs (e.g., Bloise and Johnson, 2007) may suggest age- and gender-related differences in emotional order effects, we had no specific hypotheses in this regard. Rather, our aim was to statistically control for the influence of age and gender as possible confounders of emotional order effects.

## Methods

### Sample

The sample of the present study comprised $N = 94$ older adults from the city of Ulm and surrounding areas.^3^ Participants were recruited using flyers and word-of-mouth. Their average age was 67.14 years (Range: 59–87 years, SD: 6.17 years), 48 participants (51%) were female. On a Likert-type scale (ranging from 1 = poor to 5 = excellent), participants rated their subjective health as 3.61 on average (SD: 0.85). With respect to education, 46 (49%) participants had completed Volks-/Hauptschule (equivalent to 9 years of schooling), 22 (23%) had finished Realschule (equivalent to 10 years of schooling), and 26 (28%) reported to have Abitur (equivalent to 13 years of schooling).

### Procedure

Participants were tested individually. After the participants had given their informed consent, the experiment started with the collection of demographic data (age, sex, education, subjective health) gathered via Inquisit (2015). Afterwards, participants were asked to judge their current mood on a 7-point Likert-type scale (1 = very negative ... 7 = very positive). Subsequently, participants were instructed on how the autobiographical memory task using cue words would proceed and were shown two examples of cue words and possible autobiographical memories these cue words may trigger. The instruction for the retrieval of autobiographical memories was that participants should report the “first event that comes to mind when prompted with the cue word.” Moreover, participants were asked to select memories of events as specific as possible with a clear start and ending point in time.

Next, the autobiographical memory task started, which involved the presentation of 30 cue words taken from the Berlin Affective Word List (Võ et al., 2009).^4^ Cue words were neutral in emotional quality, but high in imaginability (see the list in the appendix). They were presented on the computer screen in Arial with a font size of 40pt, the order of presentation was random, participants’ distance to the monitor was approximately 50 cm. When a participant had retrieved an AM, it was described in a few words to one of the trained research assistants conducting the experiment, who put down the content (in keywords) on a prepared answer sheet available for each AM. Also, participants were required to answer a number of questions with respect to each AM: (1) Whether they were alone or not during the event, (2) whether the event represents a singular or a repeated event, (3) how old they were when the event tok place, and (4) how vivid the AM is. These three variables will not be analyzed in the present study. Two additional questions covered the emotional intensity with regard to happiness and sadness of AMs (see below). All AM-related questions were asked and answers recorded by the research assistant on the answer sheet. Note that these questions were asked directly after participants had described an AM. The whole experiment lasted between 45 minutes and 1 hour.

### Outcome measures

Happiness Happiness of the event described in an AM was measured using the question “How happy did you feel during the event you just described?”, which participants answered on a 5-point Likert-type scale, ranging from 1 = not at all happy to 5 = very happy. In order to estimate the aggregate (between-person) reliability of the happiness ratings (cf. O’ Brien, 1990), we randomly built 5 parcels of 6 happiness ratings each and calculated the happiness mean of each parcel for each individual. Next, Cronbach’s alpha was estimated for the happiness parcel means. This procedure was repeated 500 times. The average Cronbach’s alpha of happiness across these 500 replications was 0.81.

Sadness Sadness of the event described in an AM was assessed using the question “How sad did you feel during the event you just described?”, which participants answered on a 5-point Likert-type scale, ranging from 1 = not at all sad to 5 = very sad. Using the same procedure as described for happiness, we arrived at an aggregate reliability estimate of 0.71.

Within-Person Reliability Estimation of Happiness and Sadness Ratings Regarding the happiness and sadness ratings of each AM, their correlations on Level 1 can also be considered as a reliability measure, because the same AM was judged on two nominally opposite dimensions. We used mixed-effects location scale models for both continuous (Hedeker, Mermelstein, & Demirtas, 2008) and—treating the happiness and sadness ratings more conservatively as measured on an ordered-categorical scale—ordinal outcome variables (Hedeker, Demirtas, & Mermelstein, Hedeker et al., 2009). Level 1 scale correlations were estimated as 0.88 and 0.93, respectively, showing strong within-person correlations of happiness and sadness ratings for the same AM.

### Modeling approach

If the consecutive recall of AMs is conceptualized as an associative chaining process, autoregressive models represent an adequate statistical analysis approach. With two outcome variables (happiness and sadness ratings for each of up to 30 AMs), a multivariate first-order autoregressive model AR(1) is given as

$$\begin{aligned} y_{ijk} = \beta _{0j} + \beta _{1j}y_{ijk-1} + e_{ijk} \quad (k > 1), \end{aligned}$$

where $y_{ijk}$ is the measurement of outcome variable *j* ($j = 1, 2$) in person *i* ($i = 1 \ldots N$) reported at output position *k* ($k = 2 \ldots n_i$), $\beta _{0j}$ is the intercept of outcome variable *j*, $\beta _{1j}$ is the autoregressive effect in outcome variable *j*, that is, the effect of the outcome variable measured in the directly preceding output position $k-1$, $y_{ijk-1}$, and $e_{ijk}$ is a residual. The residuals of each outcome variable *j* were assumed to be independent and normally distributed with mean 0 and (constant) variance $\sigma _{e_{jk}}^2$. The autoregressive effects can be interpreted as order effects in the recall of AMs in the sense that the intensity of happiness or sadness of an AM has an influence on which AM—more specifically, on which AM with which emotional intensity—is recalled next.^5^

The model can be extended by random effects for the intercept as well as for the autoregressive effects to capture individual differences in the general amount of recalled happy or sad AMs and individual differences in the strength of the emotional order effects. Once random intercepts and random slopes are added, the model becomes a mixed-effects autoregressive model (Rovine & Walls, 2006), which is given as

$$\begin{aligned} y_{ijk} = \beta _{0j} + u_{0ij} + (\beta _{1j} + u_{1ij})y_{ijk-1} + e_{ijk} \quad (k > 1), \end{aligned}$$

where $u_{0ij}$ is the (random) deviation of individual *i* from the fixed intercept and $u_{1ij}$ is the random deviation from the autoregressive effect in outcome variable *j*. Individual-specific random deviations from the fixed intercept and from the autoregressive effect were assumed to be normally distributed with zero means and variances $\sigma _{u_{0ij}}^2$ and $\sigma _{u_{1ij}}^2$. Moreover, random intercept and random autoregressive effects are allowed to covary with covariance $\sigma _{u_{0ij}\cdot u_{1ij}}$.

In the present study, the focus is on autoregressive effects within persons. For this reason, we centered the lag variables around their respective latent group (i.e., person) mean (cf. Gistelinck et al., 2021). The resulting mixed autoregressive model with *latent mean centering* of the autoregressive predictor variable is

$$\begin{aligned} y_{ijk} = \beta _{0j} + u_{0ij} + (\beta _{1j}+ u_{1ij})\big (y_{ijk-1}-(\beta _{0j} + u_{0ij})\big ) + e_{ijk} \quad (k > 1). \end{aligned}$$

To make this type of model with two dependent variables (happiness and sadness ratings of AMs) amenable to estimation with standard multilevel software, we introduced two indicator variables, $\delta _{i1k}$ and $\delta _{i2k}$, with the first one being 1 when the happiness rating is concerned (0 otherwise), and the second one being 1 when the sadness rating is concerned (0 otherwise) (see MacCallum, Kim, Malarkey, & Kiecolt-Glaser, 1997). An according dual mixed-effects autoregressive model with latent mean centering can then be written as

$$\begin{aligned} y_{ijk} = \sum _{j=1}^2 \delta _{ijk}\big \{\beta _{0ij} + u_{0ij} + (\beta _{1j} + u_{1j})\big (y_{ijk-1}-(\beta _{0j} + u_{0ij})\big ) + e_{ijk}\big \} \quad (k > 1), \end{aligned}$$

where all random effects are allowed to covary with covariance matrix **G**.

Of interest are not only the autoregressive effects within outcome variables, but also across outcome variables, that is, to what extent the happiness rating of the preceding AM affects the sadness rating of the current AM and vice versa. Including a crossed first-order autoregressive effect, the model becomes

$$\begin{aligned} y_{ijk}= & {} \sum \limits _{j=1}^2 \delta _{ijk}\big \{\beta _{0j} + u_{0ij} + (\beta _{1j} + u_{1ij})\big (y_{ijk-1}-(\beta _{0j} + u_{0ij})\\&+ \beta _{2j}\big (y_{i(3-j)k-1}-(\beta _{0(3-j)} + u_{0i(3-j)} \big ) + e_{ijk}\big \} \quad (k > 1), \end{aligned}$$

where the second line now contains the respective crossed autoregressive effect. For each of the two outcome variables, additional predictor variables and interactions among predictor variables can be added to the model (see Results section).

All models were estimated using SAS Nlmixed (2015). Model fit was assessed based on $-2$ times the logarithm of the likelihood ($-2\ell \ell$) of the data under the model, where smaller values denote a better model fit. In addition, we report Akaike’s information criterion (AIC), which is based on $-2\ell \ell$ but, to reward parsimony, adds a penalty factor for introducing additional parameters. Moreover, the difference in $-2\ell \ell$ of two (nested) models was used to test for a significant improvement in fit based on the approximate chi-square distribution of this difference (see Diggle, Heagerty, Liang, & Zeger, 2002). As measures of effect size, we calculated the amount of variance accounted for on Level 1 and Level 2 (see Rights & Sterba, 2019). For both levels, $R^2$ was defined as the proportion of the outcome variable variance explained on that level via fixed slopes.^6^

## Results

In total, the 94 participants reported 2792 AMs, implying that 28 (1%) AMs were missing. Because there were no intermittent missing values—missing values did always occur at the end of the sequence—, it appears likely that satiation had set in for some participants. The average number of AMs reported per participant was 29.7 (Minimum 18, Maximum 30). All analyses described below were based on a sample size of 2698 observations (= 2792 total observations − 94 observations on the first output position). As Table 1 shows, across participants and AMs, the average happiness rating was 2.98, while the average sadness rating was 2.16, indicating that participants rated AMs slightly more happy than the (theoretical) average of 2.5 and slightly less sad. Between persons, the standard deviations of happiness and sadness were 0.52 and 0.45, respectively. Within persons, standard deviations were 1.36 and 1.42 (see Table 1).

**Table 1:** Descriptive statistics of Happiness and Sadness ratings based on 2792 autobiographical memories from 94 older adults

		Between Persons			Within Persons${}^{a}$			
	Mean	Std	Min	Max	Std	Min	Max	ICC${}^{b}$
Happy	2.98	0.52	1.78	4.37	1.36	−3.23	3.00	0.12
Sad	2.16	0.45	1.13	3.33	1.42	−2.33	3.69	0.09

$^{a}$ centered within persons

$${}^{b}$$ intraclass correlation

In a first model (Model 1), fixed and random intercepts were estimated for both the happiness and sadness ratings of AMs. As Table 2 shows, the happiness intercept was higher than that of sadness^7^, implying that, overall, participants rated their AMs as substantially more happy than sad. The random intercept variances of both outcome variables were significantly different from zero, indicating reliable individual differences in the (average) happiness and sadness ratings of AMs. Intraclass correlations were 0.12 and 0.09, respectively, indicating that 12% and 9% of the total variance in happiness and sadness ratings were between persons. The random intercept correlation between happiness and sadness ratings was negative ($r = -0.50$).

In Model 2, age, sex, and current mood (all grand-mean centered) were entered as predictor variables. As can be seen from Table 2, age had a significantly negative effect on happiness ratings and a significantly positive effect on sadness ratings of AMs. By contrast, sex was associated only with the sadness ratings, which were lower (i.e., less sad) for women. Current mood was associated with both happiness and sadness of AMs in the expected directions, with participants in a more positive mood showing a higher average happiness rating and a lower average sadness rating—albeit the latter effect was significant only at $p<.10$. After accounting for individual differences in happiness and sadness of AMs ratings due to age, sex, and current mood, the correlation between random intercepts was slightly reduced. Together, predictor variables accounted for 16% of the between-persons variance in happiness ratings and for 18% of the between-persons variance in sadness ratings. Compared to Model 1, model fit was improved as indexed by the $-2\ell \ell$ and AIC fit statistics. This improvement in fit was significant ($\Delta -2\ell \ell = 21$, $\Delta$ *df* = 6, $p < .05$).

**Table 2:** Parameter Estimates of Dual Mixed-Effects Autoregressive Models (Based on 2792 Happiness and Sadness Ratings from 94 Older Adults)

	Model 1		Model 2		Model 3		Model 4		Model 5		Model 6	
	Happy	Sad	Happy	Sad	Happy	Sad	Happy	Sad	Happy	Sad	Happy	Sad
Fixed Effects												
Intercept	2.989*	2.155*	2.989*	2.156*	2.988*	2.156*	2.989*	2.156*	2.989*	2.157*	2.989*	2.157*
Age$^a$			−0.024*	0.019*	−0.024*	0.019*	−0.018*	0.016*	−0.018*	0.016*	−0.018*	0.015*
Sex$^a$ (0 = m, 1 = f)			0.057	−0.195*	0.057	−0.195*	0.055	−0.172*	0.054	−0.171*	0.044	−0.171*
Mood$^a$			0.118*	−0.067$\dag$	0.095$\dag$	−0.067$\dag$	0.069	−0.058	0.069	−0.057	0.067	−0.053
Mood$^a$ (Pos $\le$ 6)					0.067*	0.002	0.056$\dag$	0.003	0.056*	0.003	0.059*	0.004
Happy Lag$^b$							0.152*		0.152*		0.135*	−0.059*
Sad Lag$^b$								0.118*		0.119*	−0.047*	0.103*
Mood$^a$ $\times$ Lag									0.005	−0.038$\dag$	0.009	−0.025
Mood$^a$ (P$\le$6) $\times$ Lag											0.013	−0.045$\dag$
Random Effects												
Intercept Variance	0.194*	0.127*	0.163*	0.105*	0.163*	0.105*	0.122*	0.093*	0.122*	0.091*	0.123*	0.091*
Intercept Correlation	−0.503*		−0.435*		−0.471*		−0.459*		−0.466*		−0.389*	
Lag Slope Variance							0.032*	0.019*	0.032*	0.018*	0.031*	0.017*
Lag Slope Correlation							0.349$\dag$		0.444$\dag$		0.432$\dag$	
Residual Variance	1.549*	1.292*	1.549*	1.292*	1.547*	1.272*	1.493*	1.259*	1.493*	1.259*	1.488*	1.256*
Model fit												
$-2\ell \ell$	17440		17419		17414		17249		17245		17230	
$\Delta -2\ell \ell$					5		165*		4		15*	
AIC	17454		17445		17444		17289		17289		17282	
$R^2$ Level 1			0.00	0.00	0.00	0.00	0.04	0.04	0.04	0.04	0.05	0.05
$R^2$ Level 2			0.16	0.18	0.15	0.18	0.12	0.15	0.12	0.15	0.11	0.14

${}^a$centered between persons around the grand mean

${}^b$centered within persons around the latent group mean

${}^*p<.05$ (two-tailed), $\dag p<.10$ (two-tailed)

$-2\ell \ell$ = −2 $\times$ Log-Likelihood (Smaller is Better); $\Delta -2\ell \ell )$ = Difference in −2 $\times$ Log-Likelihood; AIC = Akaike Information Criterion (Smaller is Better)

$R^2$ measures were calculated according to Rights and Sterba (2019). Intraclass Correlation (ICC) of happiness ratings = 0.121, ICC of sadness ratings = 0.090

In Model 3, we examined whether the effect of current mood was constant across output positions or larger for the first few output positions. To do so, we introduced a dummy-coded predictor variable, which was 1 for output positions 2 to 6 and 0 otherwise.^8^ The interaction of this dummy variable with current mood was then included in the model. As Table 2 shows, doing so had two consequences. First, the effect of current mood on happiness ratings in all output positions was reduced, such that now both the mood effects on happiness and sadness ratings were only significant at the $p < .10$ level. Second, the interaction became significant for happiness ratings, showing that the mood effect on happiness was significantly larger during output positions 2 to 6. By contrast, the interaction effect on sadness ratings was virtually zero. Compared to Model 2, the correlation between random intercepts increased slightly (in absolute size) and the fit of Model 3 was marginally improved as indicated by the $-2\ell \ell$ and AIC fit statistics. The difference in model fit, however, was not significant. Due to the theoretical relevance of the interactions between mood and output position, we decided to continue with the interaction terms included. Together, the predictor variables introduced in Model 2 accounted for 15% of the between-persons variance in happiness ratings and for 18% of the between-person variance in sadness ratings.

**Fig. 1:**
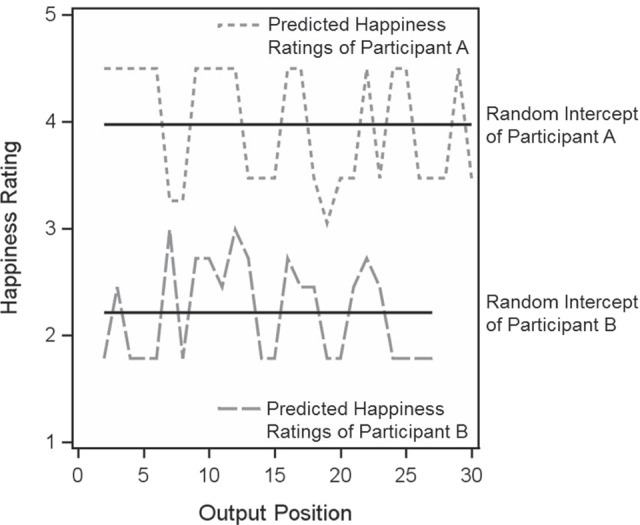
Illustration of Random Intercepts and the Autoregressive Effect in Happiness Ratings of Autobiographical Memories. Shown are the two Participants with the Highest Happiness Intercept (Participant A) and the Lowest Happiness Intercept (Participant B)

In Model 4, (latent mean centered) happiness and sadness ratings of the previous AM were included as predictors of the respective ratings of the current AM. Both were augmented by random effects. The autoregressive effect of happiness was 0.152 (see Table 2), implying that there was a tendency that a happy AM was followed by another happy AM. More specifically, because predictors are mean-centered, an AM with a happiness rating above an individual’s mean tended to be followed by another AM above an individual’s average happiness rating, while an AM with a happiness rating below an individual’s mean tended to be followed by another AM below average happiness. Figure 1 aims to illustrate this finding. In Fig. 1, the two black horizontal lines depict the random happiness intercepts of both the individual with the largest random intercept (Participant A) and the individual with the lowest random intercept (Participant B^9^). These random intercepts represent the levels of happiness of AMs the two participants are expected to tend toward. The predicted values of happiness are shown as saw-toothed dashed and dotted lines. The autoregressive effect then becomes apparent in the number of consecutive happiness ratings above or below the individual level of happiness. As Fig. 1 shows, AMs above and below an individual’s average happiness ratings tended to follow each other.

For the sadness ratings, the autoregressive effect was 0.118, which was almost as large as for the happiness ratings.^10^ Hence, similar to happiness ratings, an AM with a sadness rating above an individual’s mean (i.e., a sadder AM) tended to be followed by another AM above an individual’s average sadness rating and vice versa. Both autoregressive effects indicate that there is an emotional ordering of AMs during recall. The effects accounted for 4% of Level 1 variance in both happiness and sadness ratings. In terms of established categorisations, these effects would be considered as medium.

**Fig. 2:**
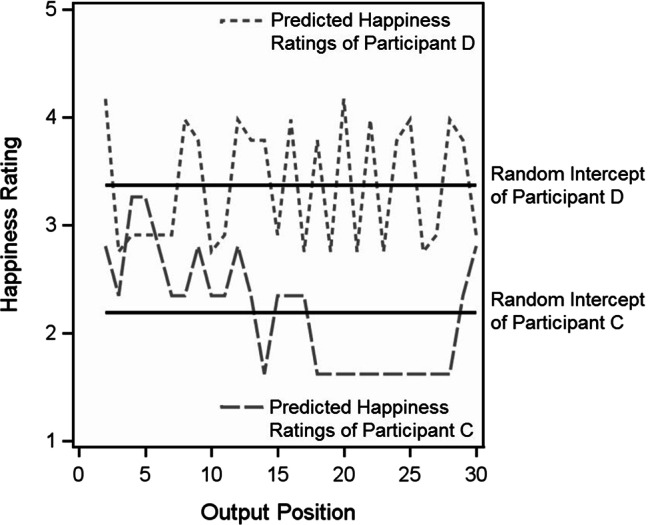
Illustration of Random Slopes in the Autoregressive Effect in Happiness Ratings of Autobiographical Memories. Shown are the two Participants with the Highest Autoregressive Effect (Participant C, 0.431) and the Lowest Happiness Autoregressive Effect (Participant D, −0.178)

For both autoregressive effects, random variances were significantly different from zero, indicating that participants differed reliably in the strength of these effects. Figure 2 depicts this finding for the happiness ratings. In Fig. 2, the predicted values of the two participants with the highest (Participant C) and the lowest (Participant D) random autoregressive effect in happiness ratings are shown. For Participant C, the autoregressive effect was estimated as 0.431, implying a strong emotional order effect, which is evident from the long sequences of happiness ratings either above or below the individuals average happiness rating. For Participant D, in contrast, the autoregressive effect was estimated as −0.178, implying a *reversed* emotional order effect—which can be seen in Fig. 2, where happiness ratings “jump” from above- to below-average across output positions. The random slopes of happiness and sadness autoregressive effects were positively correlated ($r = .35$, see Table 2), indicating that for individuals with a stronger happiness autoregressive effect, the sadness autoregressive effect also tended to be stronger. None of the other possible correlations among random happiness or sadness intercepts and random happiness or sadness random autoregressive slope was significant.^11^

After the inclusion of autoregressive effects, age and sex effects were slightly reduced. Moreover, the mood effects were no longer significant. The interaction of mood with the first few output positions in happiness ratings was also smaller than in Model 3 (and now significant only at the $p<.10$ level). Fit statistics show that Model 4 represented a large and significant) improvement compared to Model 3. The amount of explained variance on Level 1 (within-person level) was 5% for both happiness and sadness ratings, respectively.^12^

In Model 5, we examined whether current mood had an effect on the strength of the autoregressive effects. To do so, we included interaction terms between current mood with the happiness and sadness effects, respectively. The according parameter estimates can be found in Table 2. Only the interaction between current mood and the sadness autoregressive effect was significant at the $p < .10$ level, indicating that there is a tendency for participants in a more positive mood to show an attenuated effect of the preceding AM’s sadness on the sadness of the current AM. In comparison to Model 4, fit only minimally (and not significantly) improved according to $-2\ell \ell$, while the AIC indexed no change in model fit. Also, the amount of variance accounted for on Level 1 did not increase.

In a final model (Model 6), crossed autoregressive effects were added to the model as well as the interaction between mood, the first 5 output positions, and the lag effects. The effect of the happiness rating of the previous AM on the sadness rating of the current AM was $-0.059$ and significant (see Table 2), as was the effect of the sadness rating of the previous AM on the happiness rating of the current AM ($-0.047$). That is, there were autoregressive effects not only within variables, but also across variables: A sadder preceding AM reduced the happiness rating of the current AM, while a happier preceding AM reduced the sadness rating of the current AM. Hence, the output order in happiness also affected the output order of sadness and vice versa. At the same time, the within outcome variable autoregressive effects were slightly reduced, as were the random intercept and slope correlations. This finding indicates that the crossed autoregressive effects mainly (but not entirely) accounted for variance that was not explained by the within outcome variable autoregressive effects. Accordingly, happiness affects the output order in sadness of AMs, while sadness affects the output order in happiness of AMs. Regarding the interaction between mood, output positions 2 to 6, and the lag effects, we found that this triple interaction was significant at $p<.10$ for the sadness autoregressive effect. At the same time, the simple mood effect on the sadness autoregressive effect reduced to non-significance. Importantly, model fit improved significantly after the inclusion of the crossed autoregressive effects. Also, the amount of variance accounted for on Level 1 increased.^13^

## Discussion

The results of the present study demonstrate that the order with which AMs are retrieved is influenced by an autoregressive effect of the emotional intensity of AMs. Thus, we replicated the findings of Nusser and Zimprich (2021), who found an emotional valence autoregressive effect in the output order of AMs. Extending their results, in the present study two outcome variables were analysed simultaneously, happiness and sadness ratings of AMs as judged by the participants. Our analyses demonstrate that autoregressive effects come into play both *within* variables (e.g., effect of happiness of the preceding AM on the happiness of the current AM) and *across* variables (e.g., effect of happiness of the preceding AM on the sadness of the current AM), albeit the latter effects were smaller. Still, the crossed effects contributed significantly to the prediction of happiness and sadness ratings—a new and intriguing finding. That is, after older individuals recall a positive memory, they tend to retrieve a more positive (i.e., above the individual’s average) and less negative AM afterwards.^14^ Our results thus support the assumption that the voluntary recall and re-construction of a specific AM is mediated by (emotional) activations prior to the conscious experience of a memory (e.g., Conway, Justice, & D’Argembeau, 2019). This is in line with Kensinger and Ford (2020), who argued that past events accessed have an effect not only on our actions and decisions, reshape memory traces, or lead to new interpretations and evaluations of the event (e.g., Kornell & Vaughn, Kornell & Vaughn, 2016; Schwabe, Nader, & Pruessner, 2014). They can, in addition, be seen as a *starting point* describing the power of retrieval to elicit effects that continue into the future, that is, subsequent retrieval. Our results are also in agreement with the idea of emotional associations between AMs along which memories of the same valence can activate each other during recall (Bower, 1981; Philippe et al., 2009). To summarize, our findings support the assumption of an organising role of emotion in autobiographical memory (Nusser & Zimprich, 2021; Philippe et al., 2009; Philippe et al., 2011).

In accordance with the findings of Nusser and Zimprich (2021), participants of the present study differed significantly in the strength of the autoregressive effects of happiness and sadness. To account for these differences, we considered mood as an explanatory variable. However, emotional order effects were only weakly affected by the mood of participants at the beginning of the autobiographical memory task in that participants with a more positive mood reported happier AMs during output positions 2 to 6 and showed a less pronounced autoregressive effect for the sadness ratings. Note that the latter effect was significant only at the $p < .10$ level. Nevertheless, these findings have two implications. First, being in a positive mood leads to higher happiness ratings especially of the first AMs. The finding that mood-congruency for positive AMs is most pronounced at the beginning of the recall process is an important result given that most studies investigated mood-congruency by averaging emotionality across AMs (e.g., Berntsen, 2002, Exp.3; Miranda & Kihlstrom, 2005). Second, the negative effect of mood on the autoregressive effect of sadness especially during the first few output positions indicates that people in a positive mood are less prone to continuously retrieve negative memories once they recalled a sad memory. In contrast, people in a negative mood may tend to stay in a “negative cognitive loop” (Isen, Shalker, Clark, & Karp, Isen, 1978), perhaps reflecting rumination on negative experiences and disruption in the recall of positive AMs—much as it has been shown in depression and dysphoric mood (e.g., Joormann, Siemer, & Gotlib, 2007). Whereas negative mood seems to be more dysfunctional in that it reinforces the subsequent recall of negative AMs, positive mood appears to be more functional in the sense that it buffers the autoregressive effect of sadness. This may be explained by a general tendency to maintain positive mood states by, for instance, focusing attention away from negative information (e.g., Carstensen, Pasupathi, Mayr, & Nesselroade, 2000; Isen, 1987). However, as Schwager and Rothermund (2014) argued, mood is a rather diffuse state (see also Larsen, Hemenover, Norris, & Cacioppo, 2003) that “reside(s) in the background of consciousness”(p. 973). From this perspective, mood is less likely to trigger emotion regulation efforts compared to the experience of very intense emotional states—which might help explain the relatively small effects of mood we found in the present study.

In the present study, autoregressive effects were found for AMs prompted by emotionally neutral cue words and after statistically controlling for the influence of age-related and sex-related differences. We used emotionally neutral cue words, a procedure based on the assumption that so-elicited AMs result in an unbiased and representative sample of the events of a participant’s life. In line with this assumption, happiness and sadness ratings varied considerably across AMs both between and within participants—something one would not expect to observe with respect to important memories (cf. Wolf et al., 2021). For the purpose of investigating emotional order effects, the use of neutral cue words may have another advantage. The cue words we used were relatively unspecific (e.g., “cupboard”) or “generic,” which may also have resulted in a relatively unspecific or diffuse search set: One would assume that a number of different AMs are associated with the cue words. Based on the cue overload (or cue distinctiveness) principle (Watkins & Watkins, (1975)﻿—which states that if a cue is associated to many memories, it becomes harder for that cue to elicit any single memory—the memory search process activated by generic (i.e., hardly distinctive) cue words leads to a larger number of “competitive” AMs. The probability of a single AM being selected for recall may then depend on other factors than cue characteristics, one of which may, of course, comprise emotional attributes.

In autobiographical memory research, a distinction is made between direct retrieval, that is, when based on a “bottom-up” process a cue causes a pattern of activation that directly elicits an associated memory, and generative retrieval, which based on a “top-down” process entails the intentional and effortful retrieval of memories (see, e.g., Conway, 2007). Assuming that memory retrieval—in response to relatively unspecific or “generic” cue words—was mostly generative in the present study, the role of similar emotional attributes of subsequently reported AMs may also be conceptualized from a different perspective. Part of a generative retrieval process is a cycle of access, evaluate, and elaborating of cues such that potential memory cues activate each other. Uzer, Lee, and Brown (2012) refer to this cycle as “cue generation,” which is assumed to continue until, finally, a cue is activated that successfully “links” to a fitting AM in the autobiographical knowledge base (cf. Conway & Loveday, 2010). A non-conscious spreading activation due to emotional attributes may then—during the process of cue generation—increase the likelihood that an emotionally similar AM is selected for recall.

This line of argumentation leads to the question of whether the autoregressive effect would be different for AMs elicited differently. An alternative and frequently used technique involves asking for important memories. However, it appears as if the output order in the recall of subjectively important life events is affected by attributes of AMs different from their emotional intensity (Nusser, Wolf, & Zimprich, 2022). One reason for this may be that the instruction to retrieve important AMs requires a directed, strategic search process in autobiographical memory (cf. Unsworth et al., 2014). And although important memories can, of course, be also emotionally intense, ordering important AMs chronologically (whether in a forward or backward manner) seems to be the dominating principle (Nusser et al., 2022). Moreover, the output order during the recall of important AMs may be based on a task-driven retrieval effect, that is, an *ad hoc* organisation with the purpose of reporting important AMs in an intelligible way (cf. Taylor & Tversky, 1997). In contrast, the emotional output order effects found here using neutral cue word to elicit AMs are, we would argue, more indicative of the organisation of AMs and the way they are represented in memory.

### Limitations and future directions

There are possible limitations to our approach that may reduce the generalisability of our findings. For example, happiness and sadness ratings were asked directly after an AM had been reported. The rating process itself may, thus, have increased the probability of retrieving a subsequent AM that is similar in emotional intensity. Alternatively, ratings could have been gathered at the end of the autobiographical recall task with AMs being recapitulated in random order. Nusser and Zimprich (2021) did so and also found an autoregressive effect. Still, a systematic comparison of emotion ratings given at different time points of the experimental procedure would help to shed light on possible boundaries of an emotional order effect. In addition, research on variables that increase or decrease autoregressive effects by systematically manipulating, for example, the valence of cue words, may help delineate the emotional order effects reported here.

A related issue touches the measurement of emotional intensity of AMs. In the present study, participants rated happiness and sadness of AMs on two 5-point Likert-type scales. A more elaborated assessment of the intensity of positive and negative emotions associated with an AM—by administering, for example, a number of questions tapping an AM’s emotion—may lead to a more fine-graded measure, which would allow to model emotional intensity as a latent variable (with the according measurement invariance restrictions imposed, e.g., Zimprich, Allemand, & Hornung, 2006; Zimprich, Allemand, & Lachman, 2012). A drawback of doing so, however, is that participants would then face a higher load in answering questions about AMs. Answering many questions regarding each individual AM may lead into a conflict with the requirement that the number of AMs be large to reliably estimate autoregressive effects. Thus, there is a trade-off between measuring emotional features of each AM as reliably as possible versus measuring autoregressive effects of emotional features in consecutively recalled AMs as reliably as possible (cf. Gistelinck et al., 2021).

Also, third variables may have caused the autoregressive effects found in the present study. Hintzman (2016), for example, has argued that a temporal output order of AMs may not necessarily reflect temporal contiguity, but may arise due to similarity in content. The same point can be made with respect to an emotional output order, where other, non-emotional features of AMs may underlie the autoregressive effects. Although it is difficult—if not impossible—to exclude such a third-variable explanation in autobiographical memory research (where there is no experimental control of the encoding phase of AMs), such a third variable would have to be closely correlated with the emotional intensity of AMs. As such, it appears unlikely that—analogous to an alternative explanation of temporal output order effects in AMs—an overlap in AM content may have caused the emotional output order found here. For this reason, we would also argue that emotions do not come into play only after an AM is retrieved based, for example, an appraisal of the memory’s content. Rather, our results suggest that processing and then selecting a specific AM for retrieval activates the emotional attributes of this AM and that this activation spreads to the emotional attributes of other AMs similar in emotional valence and intensity, thus raising the activation level of these other AMs (cf. Bower, 1981; Philippe et al., 2009). To disentangle the effects of different AM attributes on output order (e.g., happiness, sadness, *and* content), however, more complex models are needed, which are capable of analysing three or more outcome variables simultaneously.

Extending the dual mixed-effects model utilized in present study to include more than two outcome variables is, in principle, straightforward. The indicator variable approach (cf. MacCallum et al., 1997) allows for an unlimited number of outcome variables in the same model—albeit, in practice, this would require larger sample sizes on Level 2, the level of individuals (cf Maas & Hox, 2005). Other extensions of the autoregressive model would allow for testing more complex hypotheses regarding order effects. For example, De Haan-Rietdijk et al. (2016) suggested a multilevel threshold autoregressive model that estimates individual thresholds of emotional intensity, which, once crossed, amplify or dampen the autoregressive effect.

In the present study, current mood (at the beginning of the experiment) was included as a predictor variable of between-person differences. A more in-depth examination of the interaction between mood and autobiographical recall would require repeated measurements of mood during the recall process, which would allow to treat mood as a changing within-person (or Level 1) variable. This appears more suitable to model the dynamic interplay between mood and the recall of emotional AMs in terms of an “ongoing” mood-congruence effect (Barry et al., 2004) or affect infusion (Bower & Forgas, 2000) based on a drift of internal context (cf. Talmi et al., 2019). Apart from mood, additional person-related variables can be examined to explain individual differences in the emotional autoregressive effects found in the present study. Future studies might address whether personality traits like extraversion, neuroticism, and negative affectivity—which are known to influence the ease of retrieval of positive and negative AMs Holland & Kensinger (2010)—can account for some of these individual differences. Finally, from a developmental perspective, a comparison of emotional order effects in older versus younger adults would be of interest. With respect to a temporal order effect during the recall of important memories, we have recently demonstrated marked differences between young and old adults (Nusser & Zimprich, submitted). Whether similar age-related differences can also be found regarding emotional order effects represents an open issue.

To conclude, the present study provided further evidence for an emotional order effect in the recall of AMs, thereby extending our knowledge of the organisation of autobiographical memory. Moreover, by investigating an emotional order effect for happiness and sadness ratings of AMs separately, we did not only take emotional valence but also emotional intensity into account, which together provides a richer picture and deeper understanding of the emotional order effect. The separate analysis of the autoregressive effects of happiness and sadness enabled us to demonstrate autoregressive effects both *within* and *across* the ratings of happiness and sadness, which supports the view of emotionality as a two-dimensional construct rather than a bipolar continuum. In addition, our results revealed a mood effect on the autoregressive order effect of sadness. This may indicate a functional character of positive mood on negative memory recall, which, however, needs to be replicated in future research.

## Appendix

**Table 3:** List of 30 German cue words (with English translations) taken from Võ et al. (2009) used in the present study. Rmotionality was measured on a scale from -3 (negative) to 3 (positive), imageability was measured on a scale from 1 (low) to 7 (high)

Cue Word	Emotionality	Imageability
Apfel (apple)	1.20	6.23
Bürste (brush)	0.23	5.68
Fenster (window)	1.55	6.50
Fahrrad (bicycle)	1.14	6.14
Film (movie)	1.50	5.89
Gabel (fork)	0.30	5.77
Halle (hall)	0.20	4.86
Hocker (stool)	0.14	5.36
Hotel (hotel)	1.15	5.55
Huhn (chicken)	−0.09	5.91
Keller (cellar)	0.00	6.33
Klingel (door bell)	0.14	4.73
Klippe (cliff)	0.10	5.50
Korb (basket)	0.41	5.64
Lehrer (teacher)	0.05	4.95
Leiter (ladder)	0.25	5.18
Mappe (folder)	0.30	4.50
Pullover (pullover)	1.05	5.91
Rede (speech)	0.24	5.12
Riese (giant)	0.20	5.45
Schere (scissors)	0.00	5.95
Schrank (cupboard)	0.27	6.14
Sprung (jump)	0.38	5.38
Straße (street)	0.18	6.46
Tabak (tobacco)	0.30	5.89
Tinte (ink)	0.35	5.05
Treppe (stairs)	0.05	5.95
Turm (tower)	0.27	5.64
Uhr (clock)	0.09	6.73
Winter (winter)	0.32	6.57
Mean	0.41	5.69

## Footnotes

1 The spreading activation assumption is also part of more recent models aimed at explaining false memories, for example, the associative-activation theory suggested by Howe et al. (2009; see also Otgaar et al., 2019) or the associative-monitoring theory proposed by Roediger, Balota, and Watson (2001).

2 In Bower’s (1981) model, not only emotionally similar memories become activated, but other emotion-related aspects as well, e.g., expressive behavior, autonomic reactions, verbal labels, etc. (Niedenthal, Halberstadt, & Innes-Ker, 1999)

3 Originally, the study was designed as an investigation of the reminiscence bump phenomenon in old age (e.g., Zimprich & Wolf, 2016, 2018). However, after having finished the work on a previous article (Nusser & Zimprich, 2021), we decided that the data of the present study could be more fruitfully used to examine emotional order effects.

4 The full list of cue words can be found in Table 3 in the Appendix.

5 A pertinent methodological question is how to adequately include the first observation in the sequence of measurements (initial conditions problem). In the present study, we consider the first output position as predetermined (i.e., unaffected by the autoregressive process) because, for longer sequences of measurements, the initial conditions problem only plays a minor role statistically (Gistelinck, Loeys, & Flamant, 2021).

6 In Rights and Sterba (2019) these two $R^2$ measures are abbreviated as $R_w^{2(f1)}$ and $R_b^{2(f2)}$.

7 This intercept difference was significant ($t = 10.03$, $p < .05$, Cohen’s $d = 0.64$).

8 The rationale behind choosing output positions 2 to 6 was that in order to estimate a reliable and unbiased autoregressive effect, at least four to five repeated measurements of the outcome variable are required (see Gistelinck et al, 2021). With choosing five (instead of four) repeated measurements, we were on the slightly more conservative side. There are other, more advanced ways to define interaction variables in autoregressive models (cf. De Haan-Rietdijk, Gottman, Bergeman, & Hamaker, 2016), which were beyond the scope of the present paper, however.

9 Participant B reported only 27 AMs and, consequently, there are only 27 output positions for this individual, of which 26 were used as outcome variables.

10 We tested for a significant difference between the two autoregressive effects by constraining them to be equal. The resulting parameter estimate for equal lag effects was 0.149 and model fit was almost identical to that of Model 4 ($-2\ell \ell$ = 17251$, \Delta -2\ell \ell$ = 2, $p < .15$, implying that the two lag effects in Model 4 were not significantly different.

11 The largest of these correlations (in absolute size) was the correlation between happiness random intercepts and the happiness random autoregressive slope ($r = -.11$).

12 In additional models (not shown in Table 2), we tested for interactions between age and sex with the happiness and sadness autoregressive effects. None of these interactions was significant or of any substantial effect size.

13 In an additional model (not shown in Table 2), we also treated the crossed autoregressive effects as random. The resulting variance and covariance estimates, however, were small and not significant.

14 An anonymous reviewer noted that emotional valence can be conceptualized as one bipolar dimension or as negativity and positivity of an emotion forming to separate, but related dimensions. While this issue is not fully resolved in the emotions literature (e.g., Mattek, Wolford, & Whalen, 2017), our results do not critically depend on either of the conceptualisations of the dimensional structure of valence. What we have shown is that *if* happiness and sadness are rated separately for each AM, these ratings exhibit autoregressive effects both within and across variables. Having said this, we think that separate valence dimensions are more in line with our data because within persons (Level 1) the correlation between happiness and sadness was $r = -.36$.
